# Sensitive Data Flagging within Data Quality reports: R and Regex Integration for Effective Text Flagging in Large Datasets

**DOI:** 10.23889/ijpds.v9i5.2730

**Published:** 2024-09-18

**Authors:** Alex-Ioan Coldea, Muhammad A Elmessary, Daniel S Thayer, Ieuan Scanlon, Hannah Davies

**Affiliations:** 1 Swansea University

## Objective

We’re introducing a comprehensive approach to enhance sensitive data flagging within our Data Quality (DQ) tool. Within de-identified health records, sensitive data such as user ids, post codes, phone numbers, and addresses pose significant privacy risks if exposed in their raw form. To address this challenge, we propose using all necessary regex patterns and free text field checks for sensitive data flagging with the objective of efficient detection within large datasets and ultimately reporting such data.

## Approach

A regex map has been developed along free text field thresholds, distribution counts and percentage checks to allow a generic approach. This ensures that any new regexes can be added to the flagging process without significant code changes.

Once identified, sensitive data instances, including fields that contain free text are flagged and displayed in an html report that is human readable by DQ reviewers.

## Results

Through a combination of SQL and R regex based text processing, our approach allows a seamless identification and flagging of sensitive data within datasets. Sensitive data for large tables of +25 million records with over 50 columns are getting flagged in less than 150 seconds (approx. 2 minutes).

## Conclusions

Our development offers a practical solution for sensitive data flagging in complex datasets where team members can implement robust sensitive data flagging by adding more regex formulas in the codebase.

## Implications

DQ reviewers can inspect the sensitive flagged fields in a human readable way, providing an extra measure of confidence when determining the quality of de-identified health records.

